# Knowledge and utilization of obstetric ultrasound and associated factors among pregnant mother in Africa: a systematic review and meta-analysis

**DOI:** 10.1186/s13089-025-00420-w

**Published:** 2025-03-03

**Authors:** Anteneh Gashaw, Zerihun Figa, Yonas Abebe, Abel Desalegn Demeke, Yohanes Sime

**Affiliations:** 1 Department of Midwifery, College of Medicine & Health Sciences, Dilla University, Dilla, Ethiopia; 2 Department of Nursing, College of Medicine and Health Sciences, Dilla University, Dilla, Ethiopia; 3 Department of Psychiatry, College of Medicine and Health Science, Dilla University, Dilla, Ethiopia

**Keywords:** Obstetric ultrasound, Knowledge, Pregnant women, Utilization, Africa

## Abstract

**Background:**

Obstetric ultrasound (US) is a non-invasive imaging method that employs sound waves to explore the abdominal and pelvic areas of a pregnant woman. It is recommended to have at least two ultrasound scans during pregnancy, one in the first trimester and another in the second trimester, to identify potential complications and improve perinatal outcomes. While this practice is widely implemented in developed nations, its utilization in many African countries remains suboptimal. This systematic review and meta-analysis aims to examine the level of knowledge and utilization of obstetric ultrasound among pregnant women in Africa, providing insights into its awareness and utilization across the continent.

**Method:**

A systematic review and meta-analysis were conducted following PRISMA guidelines. Extensive literature searches were carried out across various databases, including PubMed, Google Scholar, ScienceDirect, Web of Science, Scopus, and African Online Journal databases. The pooled prevalence was estimated using a weighted inverse variance random-effects model. Heterogeneity among studies was assessed using the Cochrane Q-test and I^2^ statistics, while publication bias was evaluated through a funnel plot and Egger's test. Stata v17 software was employed to analyze factors associated with the utilization of obstetric ultrasound among pregnant women in Africa.

**Result:**

A total of 622 articles were initially identified, with 23 ultimately meeting the inclusion criteria for this review, including five studies that addressed both knowledge and utilization of obstetric ultrasound. The overall knowledge level among pregnant women in Africa regarding obstetric ultrasound was estimated at 74.33% (95% CI 63.27–85.38%), while the pooled proportion of utilization was 63.3% (95% CI 51.59–75.02%). Subgroup analysis revealed that both knowledge and utilization levels were highest in Western Africa, whereas knowledge was lowest among pregnant women in Eastern Africa. Pregnant women with good knowledge of obstetric ultrasound were significantly more likely to utilize the service, with a pooled odds ratio (POR) of 8.41 (95% CI 4.66–12.16).

**Conclusion:**

This systematic review and meta-analysis revealed a moderate utilization of obstetric ultrasound among pregnant mothers in Africa, with an increasing trend over time, particularly after 2020. The overall level of knowledge about obstetric ultrasound among mothers was 74.33%, and knowledge was identified as the key factor significantly associated with ultrasound utilization.

## Introduction

Obstetric ultrasound is a non-invasive imaging technique that uses sound waves to examine the abdominal and pelvic regions of pregnant women [1]. According to the World Health Organization (WHO), it is recommended that all pregnant women undergo an ultrasound scan before 24 weeks of gestation [1, 2]. This procedure plays a crucial role in estimating gestational age, assessing placental location, identifying single or multiple pregnancies, detecting fetal abnormalities, and improving overall pregnancy outcomes [3, 4]. Additionally, ultrasounds performed when clinically necessary can enhance the accuracy of gestational age estimation, aiding in the management of potential preterm or post-term deliveries, particularly in resource-limited settings. Moreover [3, 4], it fosters emotional bonding between parents and their unborn child, often bringing a sense of joy and connection [5]. Ultrasonographic Examination in pregnant women can involve different approaches (transvaginal and transabdominal) and the use of various types of transducers (linear, curvilinear). In early gestational ages, the transvaginal approach is most commonly employed to better visualize the embryo and the formation of the gestational sac; in later trimesters, a transabdominal approach is generally preferred for optimal visualization of both fetal and maternal structures [6]. Research from Africa highlights that labor and delivery are often associated with numerous complications [4]. Sub-Saharan Africa (SSA) faces one of the world's highest perinatal mortality rates, estimated at 34.7 deaths per 1,000 births [7]. A major factor contributing to perinatal mortality and morbidity in the region is intrauterine growth restriction (IUGR) [8]. However, these risks can be greatly minimized by ensuring that pregnant women undergo at least one ultrasound scan before 24 weeks of gestation [2–4].. Ultrasound serves as a vital tool in identifying expectant mothers who are at an elevated risk early in pregnancy [7, 8].

In developed countries, ultrasound is routinely used during prenatal care. However, in Africa, there are still significant limitations on utilization of obstetric ultrasound during obstetrical cares [9, 10]. A retrospective cross-sectional study conducted among Swedish pregnant mothers reported that 97.6% underwent routine ultrasound examinations, while 33% utilized a combination of ultrasound and biochemical markers [11]. The study conducted in Adiss Ababa, Ethiopia, showed that 51.4% and 70.1% of pregnant women had good knowledge and positive attitude regarding obstetric ultrasound [12].

Research has consistently shown that women's knowledge and attitudes about obstetric ultrasound are critical determinants of their willingness to undergo ultrasound scans during pregnancy [13–15]. Women with limited awareness of the benefits of obstetric ultrasound are less likely to utilize these services and may be more resistant to their use during pregnancy [14, 15]. Therefore, it is essential to evaluate the pooled estimates of women’s knowledge, attitudes, and practices regarding obstetric ultrasound [1].

Although obstetric ultrasound plays a pivotal role in prenatal care, there is limited awareness among pregnant women in Africa about this essential imaging technique [16]. Although primary studies on the knowledge and utilization of obstetric ultrasound among pregnant mothers in Africa exist, there is a significant need for a comprehensive, continent-wide summary of evidence. This summary would provide a clearer, more holistic understanding of the general knowledge levels and the extent of ultrasound utilization across various regions in Africa. It would help identify gaps, regional disparities, and common barriers, thereby informing policy-making and improving maternal healthcare services. Such a summarized evidence base is crucial for developing targeted interventions and promoting the widespread, effective use of obstetric ultrasound, ultimately contributing to better maternal and fetal health outcomes across the continent. This systematic review and meta-analysis aims to evaluate pregnant women’s knowledge and utilization of obstetric ultrasound in Africa, highlighting regional variations and identifying opportunities for educational interventions.

### Research questions

What is the level of Knowledge and utilization of obstetric ultrasound among pregnant women in Africa?

What are the factors associated with utilization of obstetric ultrasound among pregnant women in Africa?

### Significance of the study

This study is highly significant as it compiles and analyzes existing research across Africa, providing a systematic review of pregnant women's knowledge, attitudes, and practices regarding obstetric ultrasound. By addressing critical knowledge gaps, it offers a comprehensive understanding of factors shaping perceptions, including accessibility, affordability, and cultural beliefs. The findings will inform healthcare practices and policy development, offering valuable insights for designing interventions to improve access to and utilization of obstetric ultrasound services. Ultimately, this research aims to enhance maternal and fetal health outcomes across diverse African settings.

## Methods

### Study setting and design

This systematic review and meta-analysis, conducted in accordance with PRISMA guidelines, investigated the pooled proportions of knowledge and utilization of obstetric ultrasound among pregnant women in Africa, as well as the factors influencing its use, thereby ensuring accurate and transparent reporting in the health sciences [17]. The study comprehensively synthesized research conducted across Africa, the second-largest and most populous continent, encompassing 20% of the global land area and 16% of the world’s population [18].

*Registration* this SRMA (Systematic review and Meta-analysis) protocol was registered in PROSPERO database with registration id of CRD42024542173.

### Search methods and information sources

The PROSPERO database (http://www.library.ucsf.edu/) was reviewed to identify any existing research on this topic, confirming that no ongoing or completed studies were found. A comprehensive search was conducted across several international databases, including PubMed, Google Scholar, ScienceDirect, Web of Science, Scopus, and the African Online Journal, to collect relevant data. Additionally, the reference lists and citations of published articles were examined to uncover any additional relevant studies not indexed in these databases. The search strategy utilized a combination of MeSH terms and keywords, such as: *"Knowledge" OR "Awareness" OR "Utilization" OR "Practice" AND "Associated Factor" OR "Determinant" AND "Obstetric Ultrasound" OR "Ultrasound" OR "Obstetric Imaging" AND "Pregnant Mother" OR "Pregnant Women" AND "Africa".*

### Eligibility criteria

#### Inclusion criteria

This systematic review and meta-analysis included studies that focused on the knowledge and utilization of obstetric ultrasound among pregnant women in Africa and were published in English. There were no restrictions based on race, and the inclusion period extended up to the date of last search, conducted on November 3, 2024. Observational studies, including cohort, case–control, and cross-sectional designs conducted in Africa, were considered for inclusion in this systematic review and meta-analysis (SRMA).

#### Exclusion criteria

Articles with qualitative designs and quantitative studies that did not report the outcome variables related to obstetric ultrasound knowledge and utilization were excluded from the review.

### Measurements of outcome

This systematic review and meta-analysis (SRMA) has three objectives. The first is to determine the prevalence of obstetric ultrasound knowledge among pregnant women in Africa. Primary articles reporting pregnant women's knowledge levels were included for this purpose. In all included studies, knowledge was categorized dichotomously, distinguishing between "good" and "poor" knowledge*.*. In this study, knowledge refers to the extent of understanding pregnant women have about the significance of obstetric ultrasound during their current pregnancy. This is evaluated through various questions, such as whether obstetric ultrasound is important for confirming pregnancy, determining the baby's sex, and identifying the baby's position and so on The second objective focuses on the utilization of obstetric ultrasound among pregnant women in Africa, for which we included studies involving women who used obstetric ultrasound during their current pregnancy*.*. In this study, utilization refers to the practice of undergoing obstetric ultrasound during the current pregnancy only.The third objective concerns the factors associated with the utilization of obstetric ultrasound among pregnant women in Africa. We included studies that identified factors potentially influencing ultrasound use, specifically those linked to increased or decreased utilization.

### Study selection

The process for the selection and evaluation of studies followed the PRISMA guidelines. Three authors (AG, AD, YS) independently screened and assessed the eligibility of the studies based on predefined inclusion and exclusion criteria. In cases of disagreement, a remianing two authors (YA, ZF) was consulted to resolve any conflicts. This collaborative approach ensured the rigorous selection of relevant studies and minimized potential bias. The final list of included studies was based on consensus among all authors.

### Data extraction

Using a standardized data extraction methodology developed in Microsoft Excel, each author independently extracted all necessary information adapted from the Joanna Briggs Institute (JBI) data extraction format. For the first and second objectives, a detailed data extraction format was created with multiple columns, including the first author's name, publication year, country of the study, sample size, number of mother with knowledge, those mothers who utilized ultrasound. For the third objective, Odd ratio was used. In case of discrepancies during the data extraction process, the authors re-extracted data from the primary articles collaboratively after discussion.

### Quality assessment

Since all the studies included in this SRMA were cross-sectional, the quality of each study was assessed using the modified Newcastle–Ottawa Quality Assessment Scale for cross-sectional studies [19].

### Data processing and analysis

Data extracted from each study using Microsoft Excel were transferred to STATA version 17 for formal analysis. Given the heterogeneity of the studies, a random-effects model was applied. The results were presented through, tables, text, and forest plots. To assess publication bias, Egger's statistical test and funnel plots were used, with a P-value less than 0.05 indicating significant bias. Heterogeneity was evaluated using I^2^ tests, with values categorized 0–40% as mild heterogeneity, 40–70% as moderate heterogeneity, and 70–100% as considerable heterogeneity [20]. a subgroup analysis was performed to explore potential sources of heterogeneity and provide a more nuanced interpretation, with the results displayed in a forest plot. Sensitivity analysis were performed by using leave-one-out sensitivity analysis. To identify factors associated with the utilization of obstetric ultrasound among pregnant women in Africa, meta regression was performed.

## Result

### Study selection

**Fig. 1:**
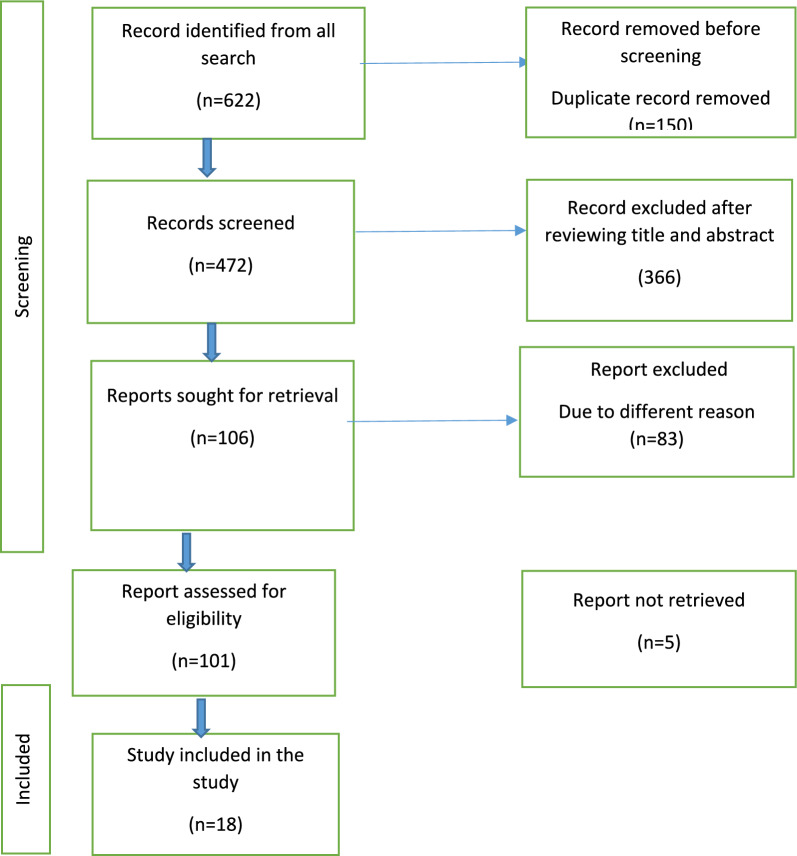
Flow chart of study selection for systematic review and meta-analysis on Knowledge and Utilization of obstetric ultrasound among pregnant mother in Africa, 2024

Our search strategy covered African Journals Online, Google Scholar, PubMed, ScienceDirect, Web of Science, and Scopus, initially identifying 622 article (608 from database and 14 from tracking citation and references from full-text paper reviewed). After removing duplicates, 472 articles remained. Title and abstract screening led to the exclusion of 307 and 59 articles, respectively. Articles excluded at the title screening stage were primarily deemed irrelevant to obstetric ultrasound or did not meet the inclusion criteria based on their titles. During the abstract screening phase, articles were excluded for reasons such as insufficient focus on the knowledge or utilization of obstetric ultrasound, lack of a relevant study population (e.g., not involving pregnant women or African settings), or methodological limitations such as being non-systematic reviews or failing to meet the quality standards required for inclusion.. From there, 106 full-text papers were thoroughly reviewed against the inclusion criteria. An additional 88 articles were excluded for various reasons: 5 articles lacked full document access, 20 had differing study populations, and 63 did not report the outcome variables, ultrasound utilization in the previous pregnancy but did not report the utilization in the current pregnancy or a qualitative study or were conducted outside Africa. Ultimately, 18 articles met the eligibility criteria and were included in the final systematic review and meta-analysis. (refer to Fig. 1).

### Characteristics of included studies

In this meta-analysis, 12 articles investigated pregnant women's knowledge of obstetric ultrasound, 11 examined its utilization, and 5 addressed both knowledge and utilization simultaneously, encompassing a total of 6,136 participants. The included studies were published between 2010 and 2024 (Gregorian Calendar).. The included studies utilized a cross-sectional retrospective study design, with sample sizes ranging from 50 to 770. Regionally, nine studies were conducted in East Africa and eight studies were in West Africa and one study from southern part of Africa. Concerning the level of knowledge about ultrasound among pregnant women, the highest prevalence was observed in Nigeria West Africa, while the lowest was reported in Ethiopia East Africa. The proportion of women with good knowledge were ranged from 35.3% to 97.6%. Regarding utilization of obstetric ultrasound the highest utilization was observed in Nigeria from West Africa and the lowest prevalence observed in Uganda from East Africa ranged from 18% to 98.3% utilization. Regarding the quality assessment of the included studies, we used Newcastle Ottawa Quality Assessment scale, The main reason for the poor quality of these articles was their failure to use robust methods to control for confounders(Tables 1, 2).

**Table 1:** Characteristics of included studies in the systematic review and meta-analysis on knowledge of obstetric ultrasound among pregnant mother in Africa, 2024

s.no	Author	Year	Region	Study design	Sample size	Mother with knowledge	Proportion	Quality
1	A. T. Agbata et al.,[21]	2018	West Africa	Cross sectional	335	326	97.6	Poor
2	B. Chinene et al.,[22]	2023	South Africa	Cross sectional	392	304	85.4	Poor
3	E. K. M. Edzie et al.,[23]	2020	West Africa	Cross sectional	384	337	87.8	Poor
4	Z. W. Haile et al.,[24]	2022	East Africa	Cross sectional	404	394	97.5	Good
5	Z. W. Haile et al.,[13]	2024	East Africa	Cross sectional	422	217	51.42	Good
6	M. Janvier et al,.[25]	2022	East Africa	Cross sectional	300	251	83.7	Poor
7	S. Loro Simon et al,.[26]	2018	East Africa	Cross sectional	50	39	78	Poor
8	B. Mengistie et al.,[27]	2023	East Africa	Cross sectional	422	165	39	Good
9	W. Molla,et al.,[28]	2022	East Africa	Cross sectional	422	148	35.3	Good
10	O. M. Oche, et al.,[29]	2013	West Africa	Cross sectional	202	195	96.4	Good
11	B. O. Usman et al,.[30]	2020	West Africa	Cross sectional	200	169	84.5	Good
12	A. Yetwale, et al.,[31]	2022	East Africa	Cross sectional	303	190	62.7	Good

**Table 2:** Characteristics of included studies in the systematic review and meta-analysis on Utilization of obstetric ultrasound among pregnant mother in Africa, 2024

s.no	Author	Year	Region	Study design	Sample size	Utilization	Proportion	Quality
1	Z. W. Haile,et al.,[32]	2023	East Africa	Cross sectional	404	284	70.3	Good
2	Z. W. Haile et al.,[24]	2022	East Africa	Cross sectional	404	394	97.5	Good
3	M. Hangelbroek et al.,[33]	2021	East Africa	Cross sectional	366	182	49.7	Good
4	O. Jagun, et al.,[34]	2013	West Africa	Cross sectional	360	245	70.3	Poor
5	M. Janvier et al,.[25]	2022	East Africa	Cross sectional	300	123	41	Poor
6	S. Loro Simon et al,.[26]	2018	East Africa	Cross sectional	50	9	18	Poor
7	C. D. Msuega, et al.,[35]	2023	West Africa	Cross sectional	250	234	93.6	Poor
8	G. K. SAMUEL et al.,[36]	2023	West Africa	Cross sectional	770	693	98.3	Good
9	A. Ugwu, et al.,[37]	2016	West Africa	Cross sectional	150	86	57.3	Poor
10	B. O. Usman, et al.,[30]	2020	West Africa	Cross sectional	200	167	83.5	Good
11	A. Yetwale, et al.,(31)	2022	East Africa	Cross sectional	303	190	62.7	Good

#### Knowledge and utilization of obstetric ultrasound among pregnant mother in Africa

**Fig. 2:**
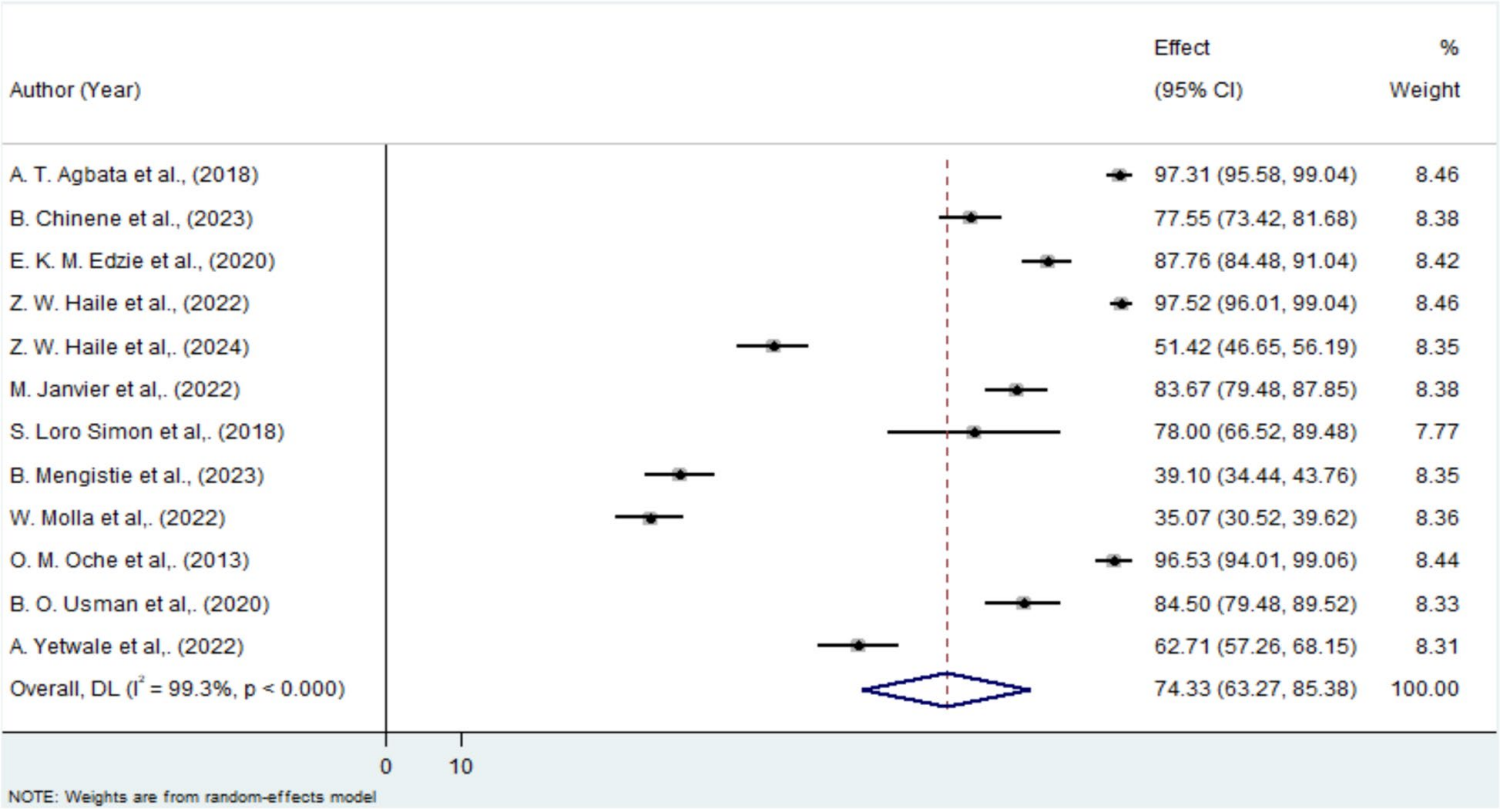
A Forest plot for knowledge of obstetric ultrasound among pregnant mother in Africa. The y axis shows the included study with corresponding publication year and the x axis shows the weighted effect from the random effect model with the corresponding confidence interval

The pooled proportion of pregnant women in Africa with knowledge about obstetric ultrasound was 74.33% (95% CI 63.27–85.38%). The Cochrane heterogeneity index indicated significant variability among the studies (I^2^ = 99.3%, P < 0.001), exceeding the 70% threshold. Consequently, a random-effects model was applied to address this variation. (Fig. 2).

**Fig. 3:**
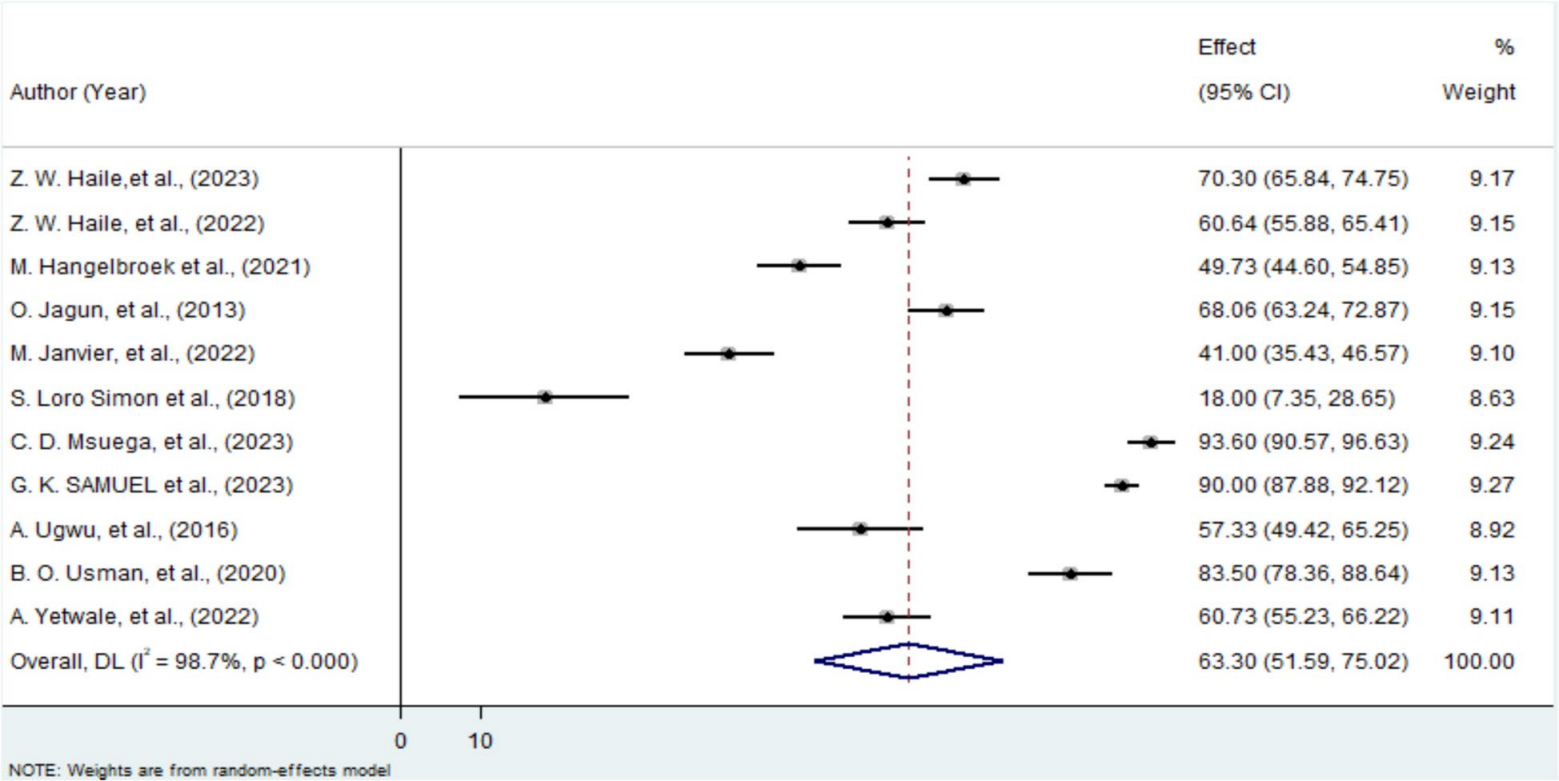
A Forest plot for utilization of obstetric ultrasound among pregnant mother in Africa

Similarly, the pooled proportion of pregnant women in Africa who utilized obstetric ultrasound during their current pregnancy was 63.3% (95% CI 51.59–75.02%). Substantial heterogeneity was again observed (I^2^ = 98.7%, P < 0.001), necessitating the use of a random-effects model (Fig. 3).

#### Subgroup analysis

**Fig. 4:**
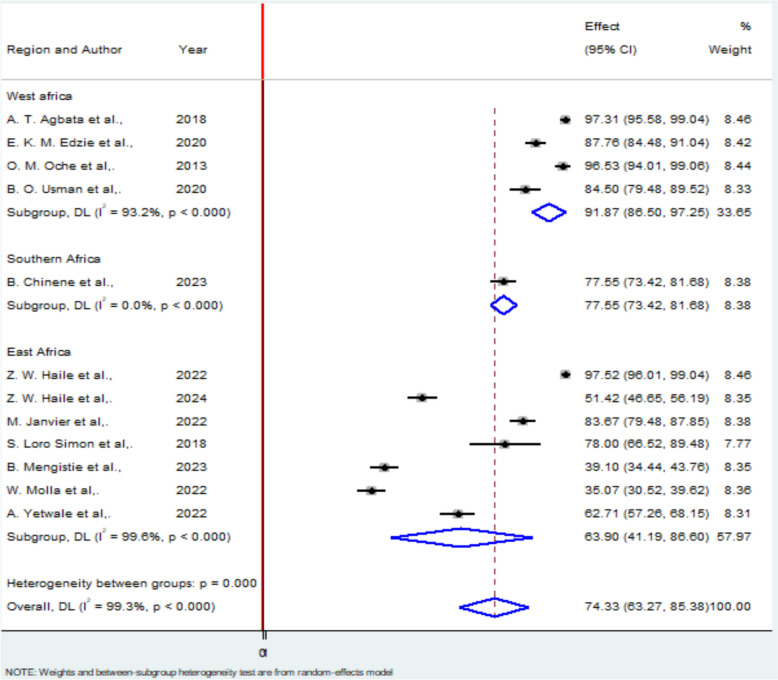
Subgroup analysis by using Region where a studies conducted among studies included in knowledge of pregnant mother regarding obstetric ultrasound in Africa. In the Y axis there is the study with regional category with their corresponding publication year and in the X axis there is the weighted effect of each region by using random effect model with their corresponding confidence interval

Following the high degree of heterogeneity we have performed a subgroup analysis based on the region where the studies were performed. The regional subgroup analysis showed that West Africa had the highest knowledge proportion at 91.87% (95% CI 86.5%–97.25%), followed by Southeast Africa at 77.55% (95% CI 73.42%–81.68%). East Africa recorded the lowest knowledge proportion, at 63.9% (95% CI 41.19%–86.63%) (Fig. 4).

**Fig. 5:**
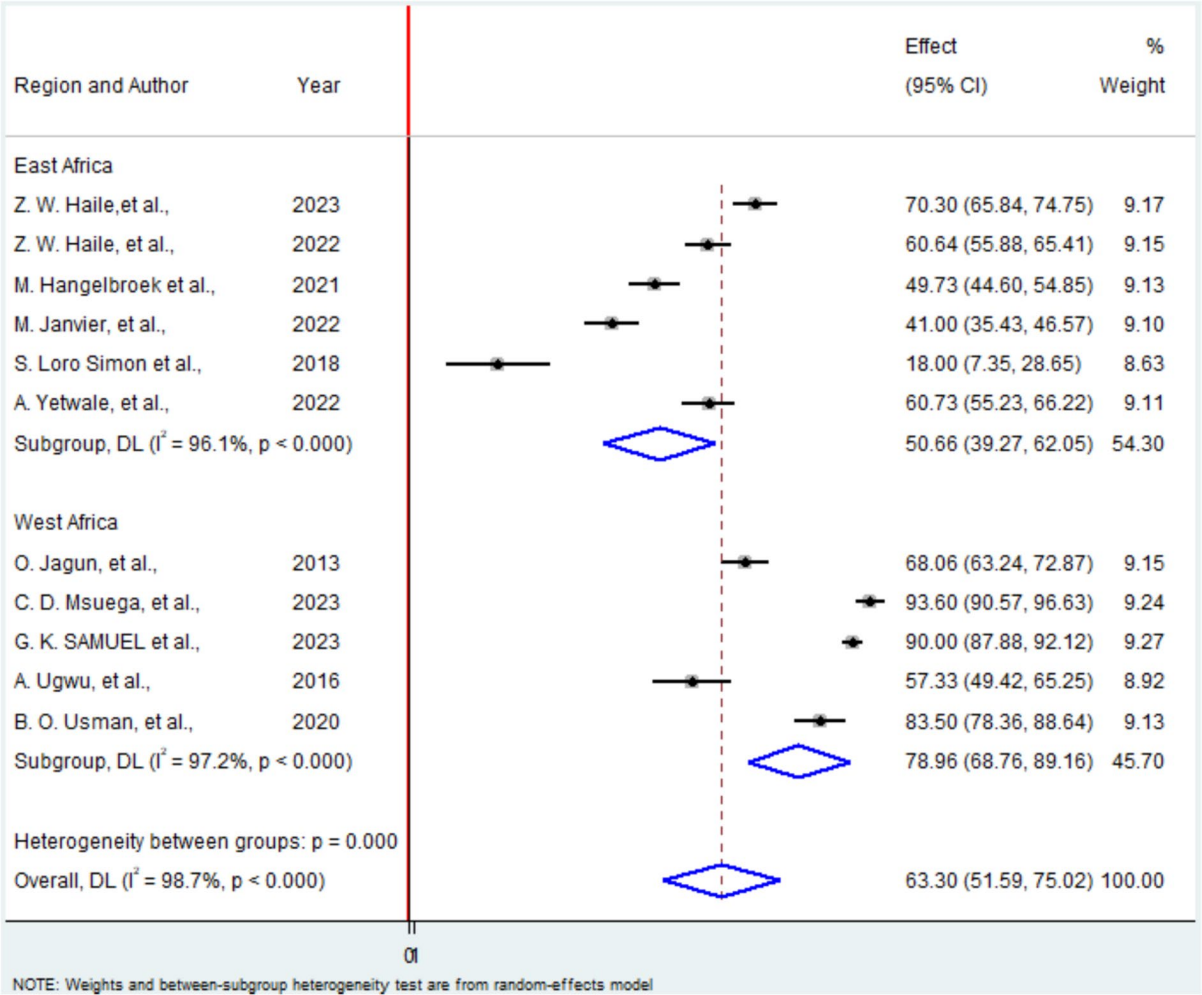
Subgroup analysis based on region where studies conducted among studies included in utilization of obstetric ultrasound by pregnant mother in Africa. In the Y axis there is the study with regional category with their corresponding publication year and in the X axis there is the weighted effect of each region by using random effect model with their corresponding confidence interval

Similarly, studies analyzing the utilization of obstetric ultrasound among pregnant women also demonstrated significant heterogeneity. Subgroup analysis by region revealed that the highest utilization rate was in West Africa, at 78.96%, consistent with the region’s high knowledge levels (Fig. 5).

**Fig. 6:**
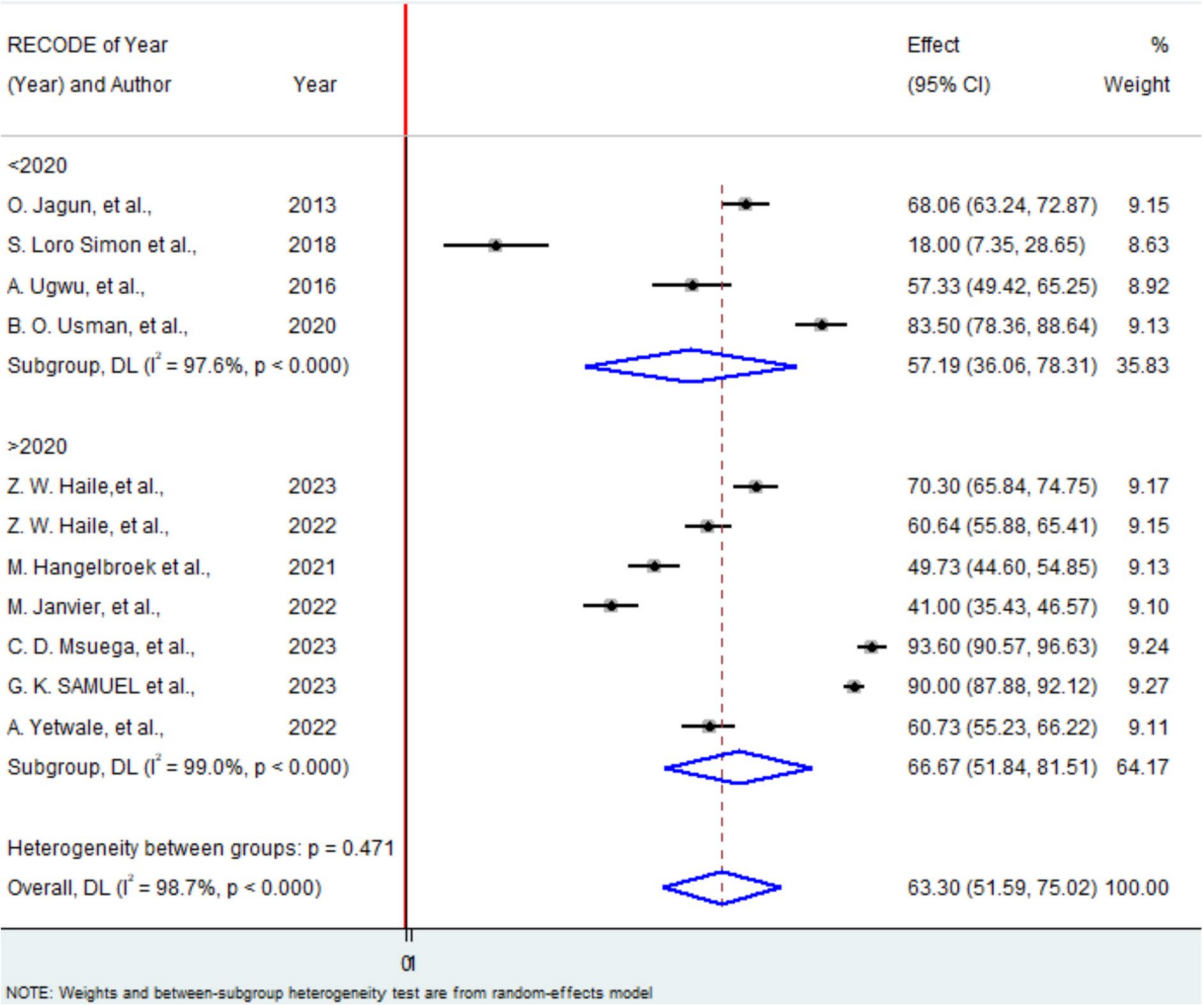
Subgroup analysis based on publication year among studies included in utilization of obstetric ultrasound among pregnant mother in Africa. In the Y axis there is the study with publication year category and in the X axis there is the weighted effect of each region by using random effect model with their corresponding confidence interval

Additionally, a subgroup analysis based on publication year indicated an increase in ultrasound utilization over time. The utilization rate among pregnant women in Africa rose to 66.67% after 2020, compared to 57.19% before that year.(Fig. 6).

#### Publication bias

**Fig. 7:**
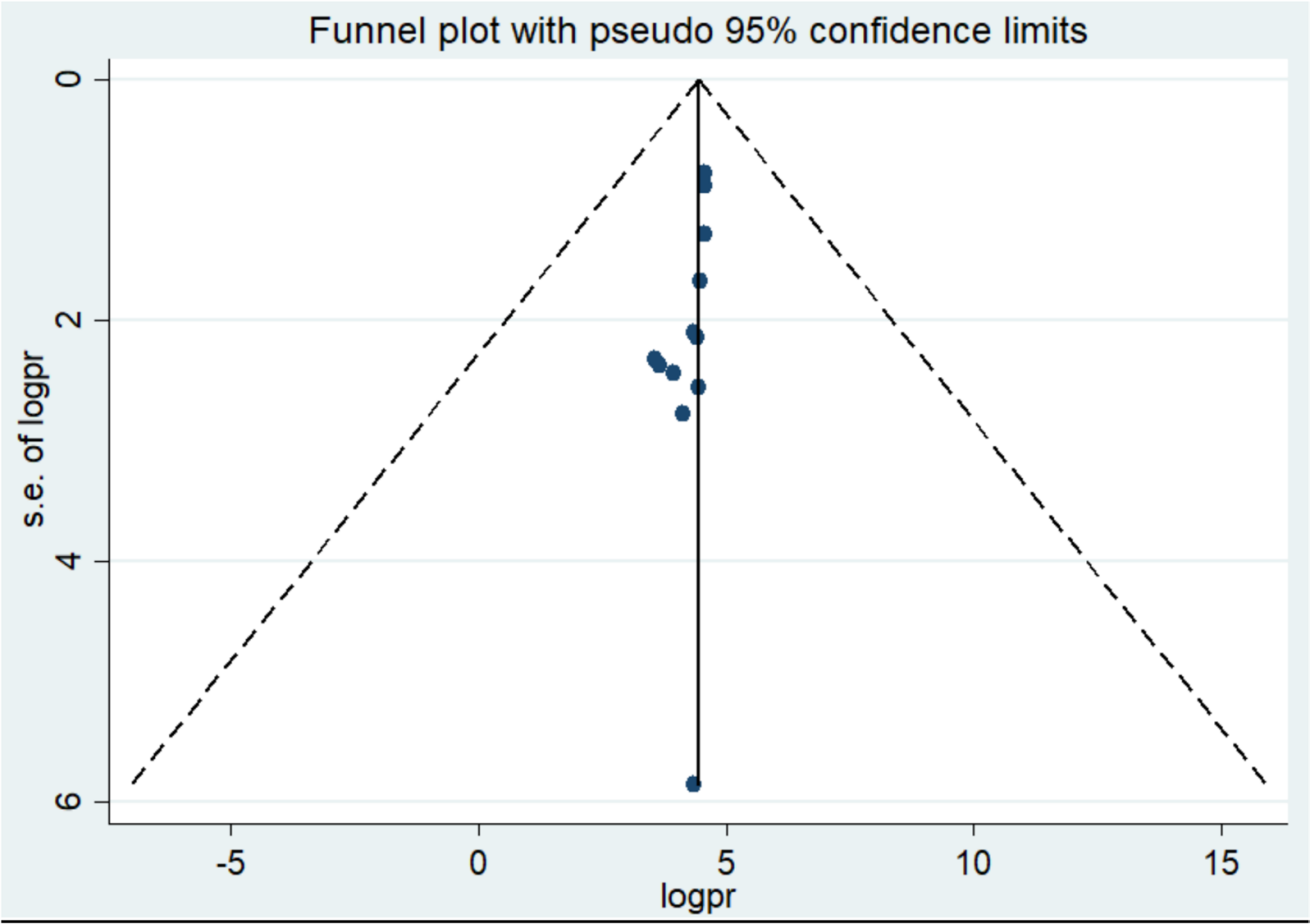
Funnel plot show distribution of studies included in knowledge of obstetric ultrasound among pregnant mother in Africa, 2024

**Fig. 8:**
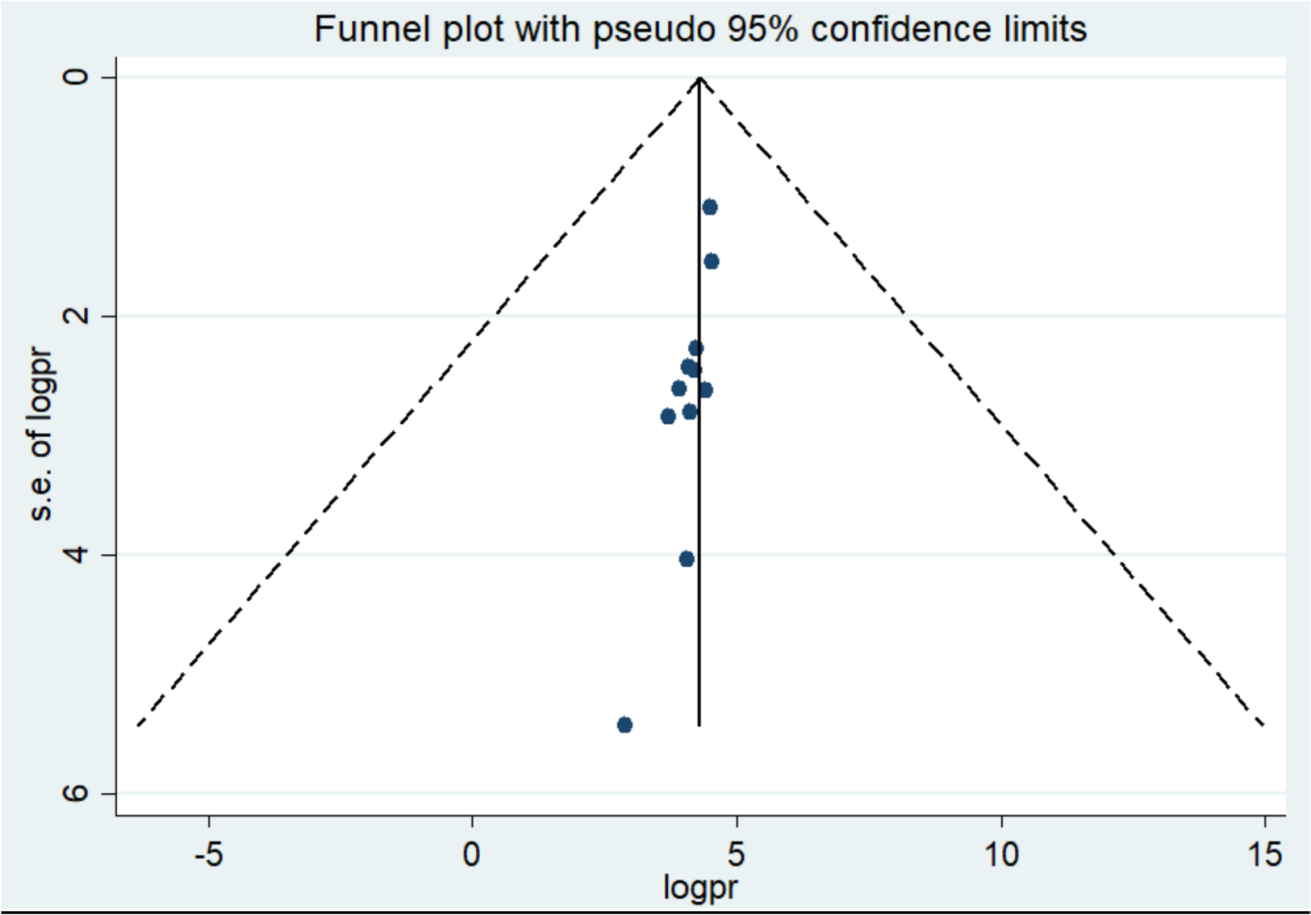
Funnel plot show distribution of studies included in utilization of obstetric ultrasound among pregnant mother in Africa, 2024

In this systematic review and meta-analysis, a funnel plot and Egger’s regression test were used to assess publication bias. For the studies examining pregnant women’s knowledge of obstetric ultrasound, the funnel plot appeared symmetrical, and Egger's test was non-significant (p = 0.1, p > 0.05), indicating no statistical evidence of publication bias. (Fig. 7). Regarding utilization of obstetric ultrasound the funnel plot asymmetical but egger test was insignificant (P = 0.2) (Fig. 8).

### Sensitivity analysis

**Fig. 9:**
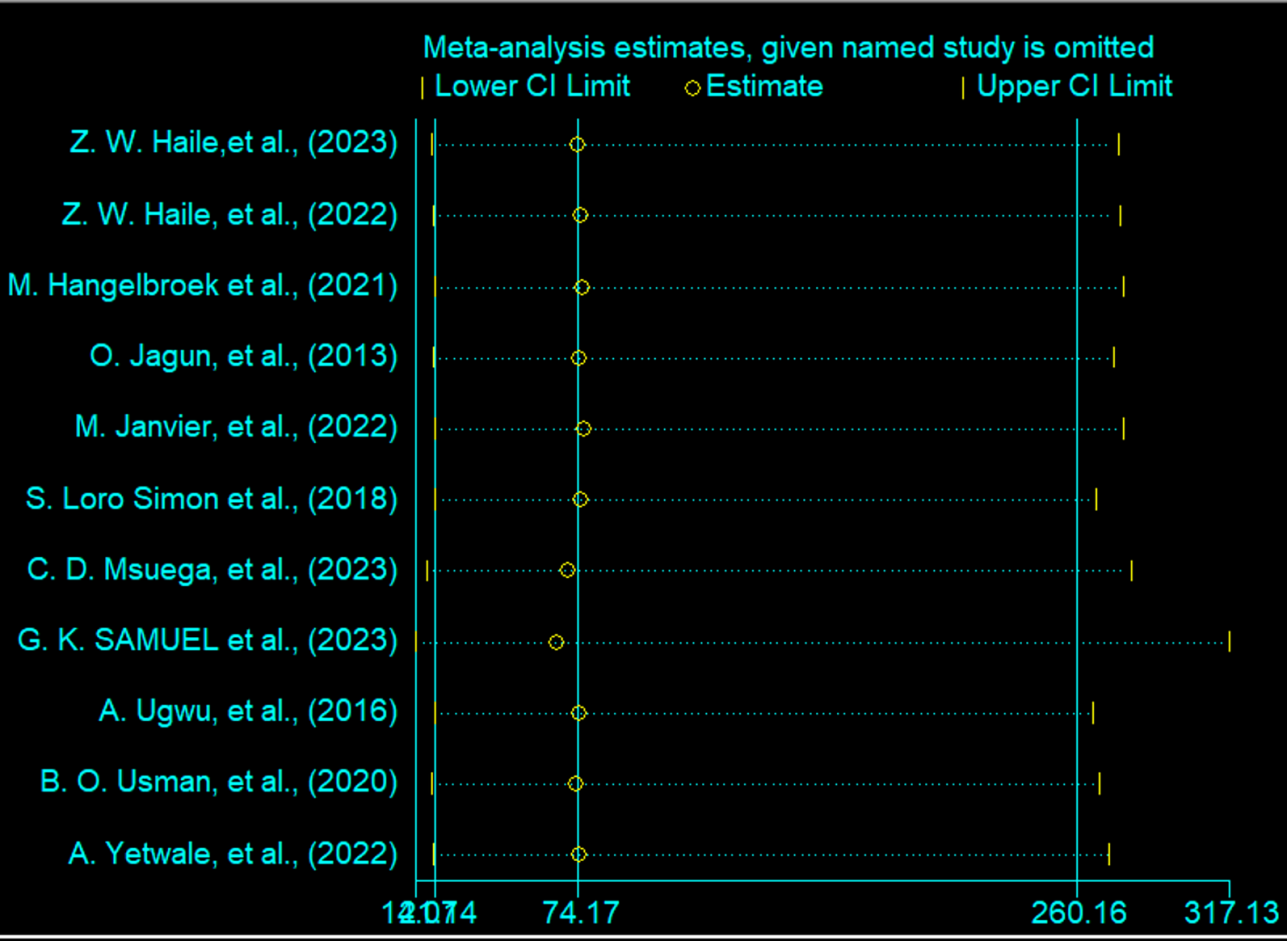
This forest plot presents a sensitivity analysis of the pooled proportion of utilization of obstetric ultrasound among women in Africa

**Fig. 10:**
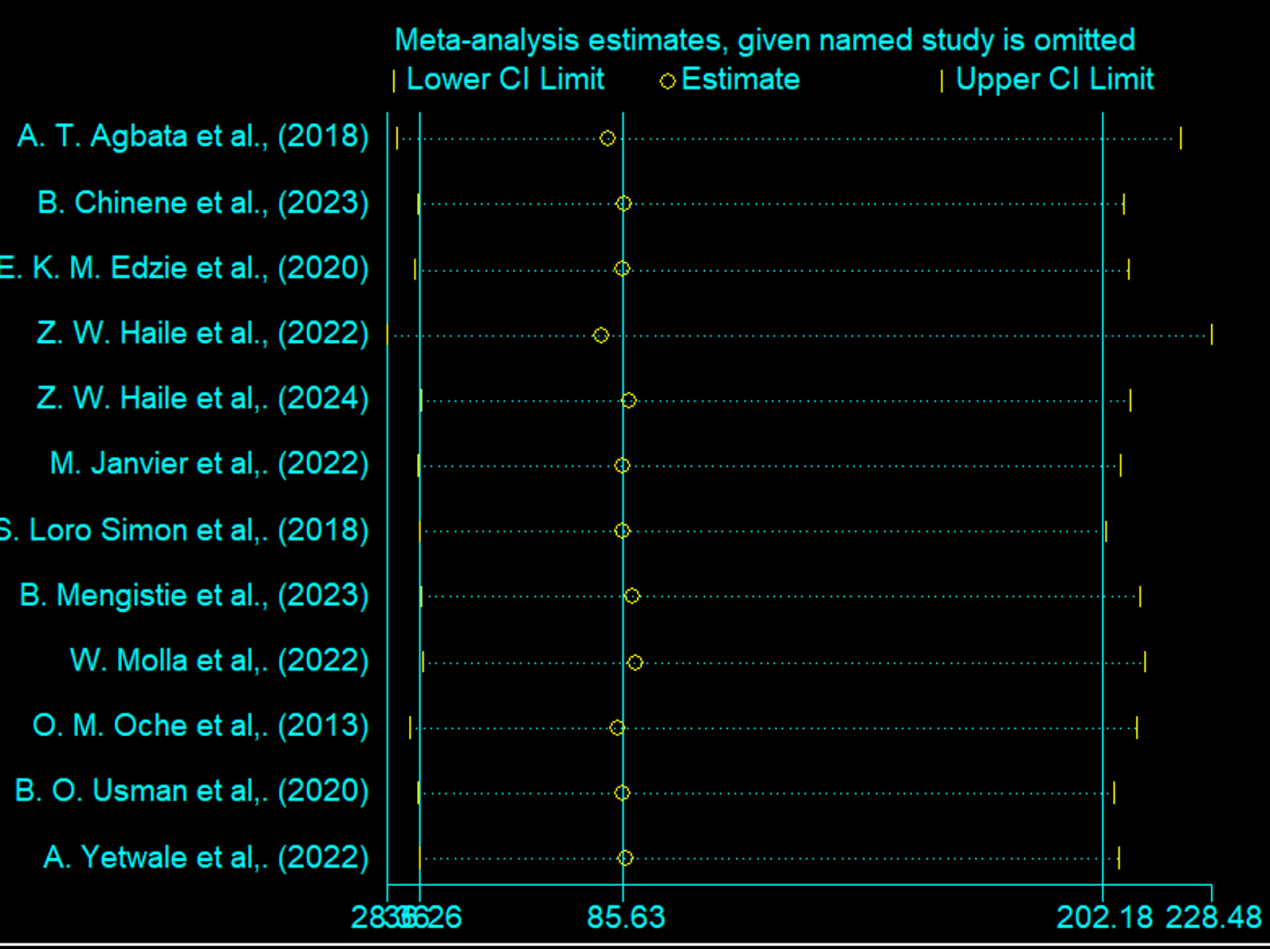
This forest plot presents a sensitivity analysis of the pooled proportion of knowledge about obstetric ultrasound among women in Africa

A sensitivity analysis was conducted to evaluate the impact of excluding individual studies on the estimated proportion of knowledge and utilization of obstetric ultrasound among women. The forest plot below illustrates this analysis, showing how the pooled proportion estimate changes when each study is omitted one by one. Each horizontal line represents the confidence interval for an individual study’s exclusion, with circles marking point estimates. The vertical line indicates the overall estimate, and the plot demonstrates that excluding any single study does not significantly alter the point estimates from the overall effect. This stability underscores the robustness and reliability of the meta-analysis findings on the proportion of knowledge and utilization of obstetric ultrasound among women in Africa (Figs. 9 and 10).

#### Factor associated with utilization of obstetric ultrasound

In our systematic review and meta-analysis; eight factors that are associated with utilization of obstetric ultrasound in two or more primary studies are included in the identification of the factors which affect the utilization of obstetric ultrasound among pregnant mother. Which includes knowledge level of pregnant mother [31, 33], being Governmental employee [24, 33], previous exposure to obstetric ultrasound [24, 33], Elementary school [24, 33], Second trimester pregnancy [24, 33], secondary school [24, 33], History of congenital malformation [31, 33] and Third trimester pregnancy [24, 33]. Accordingly, knowledge about obstetric ultrasound were significantly associated with utilization of obstetric ultrasound among pregnant mother in Africa. Those pregnant mothers with good knowledge regarding obstetric ultrasound were 8.41 times more likely to use obstetric ultrasound as compared with those mother with poor knowledge of obstetric ultrasound (Table 3).

**Table 3:** Factor associated with utilization of obstetric ultrasound among pregnant mother in Africa, 2024

Variable	Pooled AOR	95%CI of Pooled AOR
Knowledge	8.41	4.66, 12.16

Note:-

- *Pooled AOR(Adjested odd ratio)* is the point value of odds ratio when we pooled the AOR of primary studies by our analysis
- *95%CI of pooled AOR* is the 95%CI of the point value of pooled AOR that is the output of our analysis

## Discussion

Obstetric ultrasoundThe pooled level of knowledge of pregnant mother regarding obstetric ultrasound in Africa were 74.33% (95% CI: 63.27–85.38%) this finding was inline with the study conducted in southwest turkey [4] and saudi arabia [38], which may indicate similar levels of awareness about obstetric ultrasound in diverse regions, despite differences in healthcare infrastructure and socioeconomic contexts. These similarities suggest that, while knowledge levels are relatively moderate, increased ANC(Antenatal care) attendance, broader health education initiatives, and media access could significantly contribute to enhanced awareness [39]. However, this finding also highlights the potential for improvement, as roughly 26% of women in these regions still lack adequate knowledge about obstetric ultrasound, underscoring the need for targeted educational strategies to fill these gaps.

In this current systematic review and meta analyis the pooled proportiion of mother who utilize obstetric ultrasound in the currect pregnancy were 63.3% (95% CI 51.59%–75.02%). This finding is higher as compared with the study conducted in Nepal which is 26.4%[40], this might be due to those studies included in this meta analysis were more recent data reflecting advancements in healthcare delivery, while the Nepal study may focus on rural or underserved populations with limited access. Differences in the study populations and levels of education and awareness could further explain the variation. And this finding is lower as compared with the study conducted in eastern China which is 96.1% [41]. this variation also might be the population demographic factors and advanced health care delivery system in chania.

The utilization of obstetric ultrasound among pregnant mothers in Africa has shown a significant increase over time, as evidenced by the subgroup analysis based on publication years in this meta-analysis. The utilization rate after 2020 was 66.67%, compared to 57.19% before 2020. This improvement may be attributed to advancements in healthcare infrastructure, increased availability of ultrasound services, improved maternal health policies, and growing awareness of the benefits of obstetric ultrasound. Additionally, recent years may have seen greater efforts to integrate ultrasound into routine antenatal care, supported by technological advancements and training programs for healthcare providers [42, 43].

In this systematic review and meta-analysis, the only factor significantly associated with the utilization of obstetric ultrasound among pregnant mothers was their knowledge level. Pregnant mothers with good knowledge of obstetric ultrasound were 8.41 times more likely to utilize it compared to those with poor knowledge. This finding is supported by various studies [38, 41] and may be attributed to the fact that better knowledge enhances awareness of the importance and benefits of ultrasound, such as its role in monitoring fetal health, detecting complications, and planning safe delivery [32]. Increased knowledge likely empowers mothers to seek the service actively and prioritize antenatal care. Additionally, well-informed mothers may overcome cultural or societal misconceptions about ultrasound, further facilitating its utilization. This underscores the critical role of health education in improving obstetric ultrasound uptake.

Although efforts were made to minimize potential limitations in this study, it is important to interpret the findings within the context of certain acknowledged constraints. The absence of comparable reviews from other countries complicated direct comparisons, despite attempts to align with established meta-analytical outcomes. Consequently, the discussion relied more heavily on observational data, drawing from primary studies to provide a clearer understanding of the context. Aadditionally a significant proportion of the studies included in this analysis were classified as poor quality, which may limit the reliability of the findings. Additionally, there was a notable lack of studies from North Africa, with only one study from South Africa, potentially reducing the generalizability of the results to the broader African continent. Moreover, very few studies were published before 2020, which could introduce biases due to the rapid changes in healthcare practices and technology in recent years.

## Conclusion

This systematic review and meta-analysis revealed a moderate utilization of obstetric ultrasound among pregnant mothers in Africa, with an increasing trend over time, particularly after 2020. The overall level of knowledge about obstetric ultrasound among mothers was 74.33%, and knowledge was identified as the key factor significantly associated with ultrasound utilization. Those mothers possessing good knowledge are substantially more likely to use the service. These findings highlight the critical role of health education and awareness in promoting obstetric ultrasound utilization. Strengthening maternal health policies and integrating educational interventions into antenatal care programs could further enhance uptake, contributing to better maternal and neonatal outcomes across the continent.

## Data Availability

The findings of this Systematic Review and Meta-Analysis (SRMA) were derived from the collected data and analyzed according to the specified methods and materials. All pertinent data are comprehensively presented within the paper.

## Abbreviations

AOR: Adjusted odd ratio
PRISMA: Preferred reporting items for systematic reviews and meta-analyses
SRMA: Systematic review and meta-analysis
